# The Nexus of Diet, Gut Microbiota and Inflammatory Bowel Diseases in Dogs

**DOI:** 10.3390/metabo12121176

**Published:** 2022-11-25

**Authors:** Soufien Rhimi, Aicha Kriaa, Vincent Mariaule, Amel Saidi, Amandine Drut, Amin Jablaoui, Nizar Akermi, Emmanuelle Maguin, Juan Hernandez, Moez Rhimi

**Affiliations:** 1 Microbiota Interaction with Human and Animal Team (MIHA), Micalis Institute, AgroParisTech, Université Paris-Saclay, Institut National de Recherche pour l’Agriculture, l’Alimentation et l’Environnement, 78350 Jouy-en-Josas, France; 2 Oniris, Department of Clinical Sciences, Nantes-Atlantic College of Veterinary Medicine and Food Sciences, 44300 Nantes, France

**Keywords:** canine inflammatory bowel disease (IBD), diet, gut microbiota, holobiont

## Abstract

Canine inflammatory bowel diseases (IBD) are of increasing interest in veterinary medicine. They refer to complex and debilitating conditions of dogs’ gastrointestinal tract. Although little evidence for causal inferences is currently available, it is believed that IBD pathophysiology entails intricate interactions between environmental factors, the intestinal immune system, and the microbial communities that colonize the gut. To better understand the mechanisms underlying these disorders, leveraging factors associated with the development of these diseases is imperative. Of these factors, emerging evidence supports the role of dietary patterns as key players influencing the composition and function of gut microbes, with subsequent effects on health and disease. In this review, we particularly focus on addressing IBD in dogs and discuss how specific nutrients may elicit or relieve gut inflammation. Gaining mechanistic insights into such interplay and the underpinning mechanisms is key to inferring dietary recommendations, and setting up new and promising therapeutics.

## 1. Introduction

As we continue to place increasing focus on our health and wellbeing, this mindset is reflected in our pets’ lives. Inflammatory bowel diseases (IBD) are multifactorial and debilitating diseases featuring a chronic immune response, the disruption of intestinal homeostasis, and the altered composition and function of the gut microbiota, referred to as dysbiosis [1,2]. Evidence has shown that even short-term dietary changes may influence gut microbiota composition. Bacterial shifts are likely to be observed in humans within 1–3 days of extreme dietary changes, such as switching from an all-meat to an all-plant diet, being introduced [2]. A few similar studies have been performed to explore the effects of dietary interventions on canine gut microbiota composition and function [3,4,5,6,7]. Most of these studies in dogs only assess the microbial composition changes after a diet adaptation period of 10 days on average, preventing the detection of earlier changes. As dysbiosis has been linked to chronic intestinal inflammation in dogs [8,9,10,11], evidence suggests the role of diet in managing the disease. The usefulness of dietetics has been known for a long time in the treatment of IBD in dogs, particularly for the modulation of digestibility and the control of immune reactions [12]. Epidemiological data in humans and pets, and studies in rodent models have shown that low-fiber diets and food additives are likely to compromise the intestinal barrier function and contribute to a myriad of metabolic or inflammatory disorders, including inflammatory bowel diseases (IBD) [13,14,15,16]. Recently, the pet food industry has seen significant shifts and growth [17]. Indeed, the global pet food market value is expected to reach 118.83 billion United States Dollar (USD) in 2025, growing by 5.4% in the period of 2021 to 2025 [17]. Notable trends include the emergence of high-protein diets based on new protein sources, high insoluble fiber diets, raw meat diets, as well as insect-based pet food products that have made their way into the market [18]. Such schemes have been shown to impact gut motility and shape the gut microbiota, thereby influencing the overall health of the host [19,20,21]. Gut microbiota is factually known to play key roles in maintaining gut physiology [22,23]; it comprises a highly complex community that evolves and adapts to its host over the life course, and shows remarkable plasticity to environmental changes, particularly to diet [24,25].

Diet may act as a risk factor when unbalanced or highly processed, but also as a disease management strategy for gastrointestinal (GI), renal or dermatological diseases [26,27]. More recently, the relationship between diet, microbiota, and gastrointestinal inflammation has emerged as a challenging area of research. Therefore, we aim in this review to discuss dietary interventions in dogs, with a scope focused on a better understanding of the dietary-microbiota interplay in IBD.

## 2. Gut Microbiota in Canine IBD

Growing evidence suggests that bacteria present in a dog’s gut may play an essential role in its health and disease [28]. The gut microbiota of healthy dogs is known to comprise three main phyla: Fusobacterium, Bacteroidetes, and Firmicutes [29]. Within this core bacterial community, several taxa are members of the phylum Firmicutes, including bacilli and clostridia, most of which are short-chain fatty acid (SCFA) producers, such as *Faecalibacterium* spp. [30,31]. Bacteroidetes is another prominent phylum and includes the genera *Bacteroides* and *Prevotella* [32]. Similarly, the phylum Fusobacterium has been commonly associated with health in dogs [32].

Key roles of the gut microbiota include protecting against pathogens, shaping the immune system, and providing beneficial metabolites to host epithelial cells through fermentative reactions [28]. Microbial metabolites may influence host health, gut microbes, and multiple interacting communities, thereby maintaining the holobiont symbiosis [33]. They provide other beneficial effects, notably, immunomodulatory, anti-diarrheal and regulatory effects of GI motility [34]. Gut microbiota is also involved in the metabolism of bile acids (BA) as potential mediators linking gut bacteria to metabolic and inflammatory disorders [28].

Links between gut microbiota composition/function and a myriad of diseases have been widely reported. In fact, it was demonstrated in mice that gut microbiota causes several pathologies, including obesity and dyslipidemia [35,36]. Evidence suggests that microbial ecosystem imbalance or dysbiosis has been correlated with several inflammatory diseases in dogs, such as IBD [37]. Intestinal dysbiosis in dogs with IBD is often characterized by a decrease in bacterial richness and diversity [30]. Metagenomic analyses have highlighted a lower abundance of Firmicutes, while Proteobacteria increases in dogs with IBD compared to dogs with a healthy status [38]. The abundance of *Faecalibacterium* spp. and *Fusobacterium* spp. were also significantly decreased in dogs with IBD relative to healthy controls [39]. In addition, higher abundances of adherent and invasive *Escherichia coli* (AIEC) were noted in colonic biopsies from dogs with granulomatous colitis, thus highlighting a potential link with gut inflammation [40]. Metabolic alterations have also been reported, including impaired short chain fatty acids (SCFAs) and tryptophan metabolites production, which may influence intestinal homeostasis and immunological tolerance [41,42]. SCFAs (i.e., acetate, propionate and butyrate) are the main end products of intestinal bacterial fermentation of non-digestible food components, such as dietary fiber. Lower levels of acetate and propionate were detected in fecal samples from dogs with IBD compared to healthy subjects [43]. These SCFAs are known to hold therapeutic promises in IBD as they improve epithelial barrier integrity and alleviate gut inflammation in vivo. In addition to SCFAs deficiency, an altered BA metabolism has been demonstrated in canine IBD [44]. The conversion of primary BA to secondary BA is largely known to be achieved by gut microbes. BA play key roles in the emulsification and absorption of dietary lipids and serve as potent signaling molecules that act through the farnesoid X receptor (FXR) and Takeda G-protein-coupled receptor 5 (TGR5). By activating FXR and TGR5, BA can influence a variety of processes, including inflammation and lipid, glucose and energy metabolism. Accordingly, changes in gut bacterial populations have been suggested to influence inflammatory parameters and pathways through changes in BA metabolism [28,45]. In dogs with IBD, the decrease in the abundance of *Clostridium hiranonis*, a potent BA converter, is correlated with the alteration of the BA metabolism. Conversely, treatment of intestinal inflammation is accompanied by an increase in the abundance of *C. hiranonis* and a normalization of the BA metabolism [39]. The links between dietary interventions, SCFAs, BA metabolism and canine inflammation are yet to be explored. Similarly, the relevance of BA as potential therapeutic targets in dogs would need to be thoroughly addressed as investigations related to this field are still in their infancy.

## 3. Diet-Microbiota Interactions in Canine IBD

### 3.1. Dietary Proteins

Diets with high protein levels were associated with a modification of the gut microbiota composition in healthy beagles, mainly characterized by an increase in the genus *Lactobacillus* abundance. This change was linked to high concentrations of butyrate in dogs that were fed a high-protein diet [46]. Furthermore, a high protein diet has been shown to promote the growth of *Clostridium perfringens* and to reduce the abundance of *Clostridium* cluster XIVa (also known as the *Clostridium coccoides* group) in a similar population of healthy dogs [47]. Although the findings from these studies suggest that high-protein diets exert significant effects on the canine gut community, as they elicit the growth of select *Clostridium* species, a major limitation of such trials is the relatively small size of the studied cohort, limited to only twelve and nine Beagles, respectively [46,47]. Other significant differences were observed in the microbiota composition, with a higher Firmicutes:Bacteroidetes ratio in response to a high protein-low carbohydrate (HPLC) diet when compared with a low protein-high carbohydrate (LPHC) diet. Several taxa became detectable in response to diet, such as *Lactobacillus ruminis*, which was detected in 59% of LPHC-fed dogs [48]. In another study, the fecal microbiota of dogs fed a HPLC diet showed an increased abundance of Bacteroidetes in addition to an enrichment in the phylum Firmicutes [49].

In addition to the protein content of food, the protein type (origin, quality) also deserves to be evaluated. To date, few studies have addressed this question. Analysis of the impact of a hydrolyzed soy protein diet combined with oral prednisone on the gut microbiota of dogs with IBD reveals an increase in lactobacilli, *Bifidobacterium* spp., *Faecalibacterium* spp. and *Streptococcus* spp. abundance. This modification of the microbial communities is associated with an enhancement of the intestinal barrier function by increasing mucosal epithelial apical junction protein (AJP) expression [50]. Further studies are required in order to evaluate the role of dietary proteins (content, type, quality) in modeling the gut microbiota, as well as their effect on dog’s health, particularly in the context of IBD.

### 3.2. Dietary Tryptophan and Indole Derivatives

In humans with IBD, reduced availability of tryptophan or tryptophan metabolites has been suggested to contribute to the disease [51,52]. Tryptophan represents a precursor of several microbial and host metabolites, including serotonin and vitamin B3 [53]. Tryptophan metabolites are known as one of the most important endogenous ligands of the aryl hydrocarbon receptor (AhR), a nuclear protein involved in the regulation of gene expression and in maintaining intestinal homeostasis [54]. Microbial metabolites or dietary factors may influence this pathway.

Dogs with IBD and dogs with protein losing enteropathy have also been shown to exhibit lower plasma tryptophan levels than healthy dogs [55,56]. While these studies highlight a potential role of tryptophan in dogs with IBD and protein losing enteropathy, the small cohort size (10 dogs) in the IBD study and the retrospective study design for the protein losing enteropathy pathology represent major limitations. Further prospective studies with larger cohorts are needed. Further analysis of dog’s gut microbiota would be beneficial to such studies as the link between the decrease in the plasma concentration of tryptophan and dysbiosis is not yet established. In addition to the functional abnormality of the intestinal microbiota, an absorption defect linked to intestinal inflammation could also be involved.

### 3.3. Dietary Fibers and SCFAs

**Figure 1:**
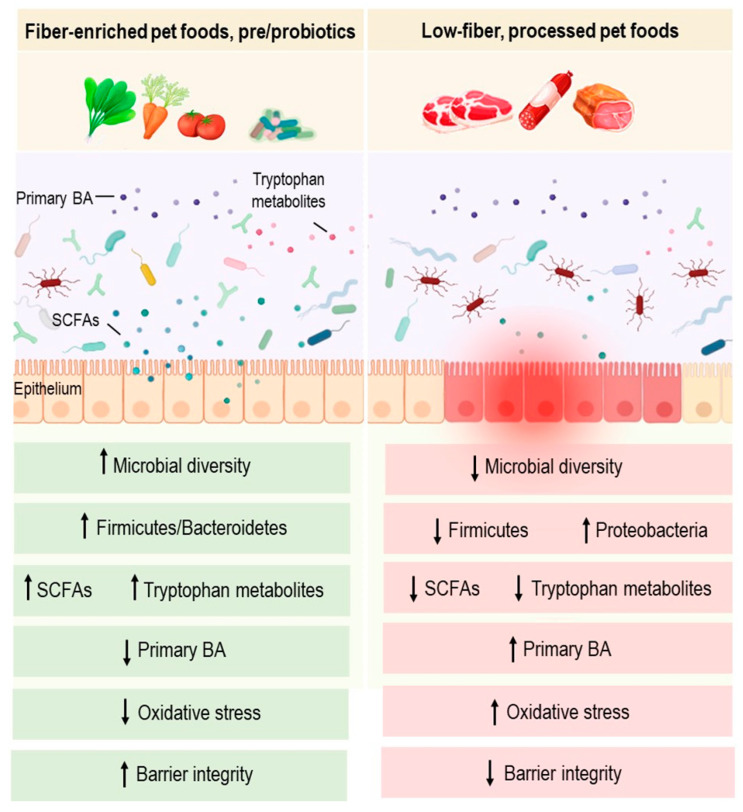
Overview of anti- and pro-inflammatory influences of dietary interventions in dogs. Dietary patterns are linked with changes in the composition and function of the gut microbiota in dogs. Up and down arrows represent increases and decreases, respectively. SCFAs: short-chain fatty acids, BA: bile acids.

Fibers can be defined as non-digestible carbohydrates that come from plants. They can be classified according to their solubility or fermentability. Soluble or fermentable fibers, such as pectin, gum Arabic, and fructooligosaccharides, support normal GI microflora growth and provide fuel for colonocytes. Several human studies showed that they also delay gastric emptying and inhibit absorption in the small intestine [33]. Insoluble fibers, such as cellulose and oat fiber, were shown to increase the volume and water content of stools, to absorb toxins and to normalize colonic motility [33]. SCFAs, including butyrate, acetate and propionate, are well-studied microbial metabolites primarily produced by the bacterial fermentation of non-digestible dietary fibers. Thus far, most human clinical trials investigating the anti-inflammatory effects of dietary fibers have been linked with a higher luminal production of SCFAs following the intake of high-fiber foods [57,58]. It is well demonstrated that SCFAs not only contribute to the regulation of the mucosal barrier function but also provide immune regulatory functions [33]. In addition, their production provides an acidic luminal environment that inhibits the proliferation of pH-sensitive pathogenic bacteria such as Enterobacteriaceae [59]. Furthermore, in human studies, SCFAs are likely to modulate inflammation by increasing the production of anti-inflammatory cytokines, decreasing pro-inflammatory cytokines, and activating the transcription factor Foxp3 [60]. Studies applied to dogs in this regard are still in their infancy and few reports have explored the role of fiber-enriched diets in canine IBD. Interestingly, the intake of high-fiber diets has recently been shown to alleviate acute large-bowel diarrhea and to exhibit significant clinical benefits in dogs [61] (Figure 1). However, the use of antibiotic and antiparasitic treatments and the absence of microbiota analysis are important limitations in this study. More controlled studies are therefore required to confirm these effects. Further metagenomic analysis of dogs’ gut microbiomes would shed light on the functional potential of this community, and provide mechanistic knowledge linking dietary fiber, gut microbiota, and the treatment of canine IBD.

The characterization of fecal fatty acids in dogs with IBD has highlighted alterations in SCFAs profiles as well as in gut microbiota composition [34]. A significant decrease in the fecal concentrations of acetate and propionate is demonstrated in the IBD group compared to the control group. A correlation between the decrease in these SCFAs and the abundance of the Bacteroidetes, *Fusobacterium* spp., *Faecalibacterium* spp., *C. hiranonis*, *Blautia* spp., *Streptococcus* spp., Ruminococcaceae, *Bifidobacterium* spp., *C. perfringens*, and *E. coli* is reported by authors [34]. Most of these bacteria are known for their fermentation capacities and as primary (Bacteroidetes) or secondary (*Blautia* spp., *Faecalibacterium* spp.) fermenters of carbohydrate. Mechanistic studies that link changes in gut bacteria composition, SCFAs production and canine intestinal inflammation have yet to be performed.

### 3.4. Dietary Fat and Bile Acids

Given their direct relationship with intestinal microbiota, BA are promising therapeutic targets in dogs with IBD. Primary BA are synthesized by the liver from cholesterol and conjugated to the amino-acids glycine or taurine. They are further subjected to deconjugation by gut microbes via bile salt hydroxylase (BSH) enzymes and dehydroxylation to yield secondary BA [28]. Higher fecal levels of primary BA were detected in dogs with IBD and correlated with a lower expression of apical sodium-dependent BA transporter proteins (ASBT) in the ileum [62]. This impaired absorption of primary BA due to ASBT downregulation was further suggested to directly contribute to diarrhea in dogs with IBD [62]. The gut microbiota is the sole metabolic pathway for BA metabolism. Thus, intestinal dysbiosis with a decrease in bacteria bearing BSH activity may imply lower BA deconjugation and dehydroxylation. In dogs, a significant decrease in the fecal abundance of *C. hiranonis* is reported during chronic inflammatory enteropathy. Known for its ability to convert primary BA into secondary BA, *C. hiranonis* may illustrate the link between dysbiosis and intestinal inflammation [63].

Interestingly, fat can be considered a nutrient of concern in some dogs with IBD. Fat malabsorption or reduced fat digestion may lead to an increased passage of fat into the colon, which can be associated with dysbiosis as well as increased intestinal permeability [64].

### 3.5. Vitamins

Reduced serum concentrations of several vitamins, including folate and cobalamin, have been reported in dogs with IBD. Vitamins are known to play a pivotal role in several cellular processes, such as GI epithelial cell turnover and repair [65]. Folate and cobalamin (also referred to as vitamin B12) are both essential water-soluble vitamins for dogs. Vitamin B12 is a component of coenzymes and is essential for cell biosynthesis and metabolism in vivo. Vitamin B12 is primarily absorbed in the terminal ileum. Most commercial pet foods are supplemented with cobalamin; nevertheless, dietary cobalamin levels vary among diets [66]. Lower serum levels of cobalamin have been reported in canine IBD as well as in exocrine pancreatic insufficiency and ileal malabsorption [67,68]. Previous reports have linked reduced serum vitamin B12 concentrations with a higher abundance of *E. coli* and enterococci, which gave rise to one of its clinical uses, highlighting it as a marker of dysbiosis [67,69]. In addition, hypocobalaminemia is a negative prognostic factor in dogs with IBD [70]. Folate is primarily absorbed in the duodenum and proximal jejunum and is synthetized by a variety of commensal bacteria. Folate may be increased in the serum of dogs with IBD. It is believed to be the consequence of the proliferation of folate-producing bacteria and to reflect dysbiosis [69]. However, more comprehensive metabolomic studies are needed to elucidate their contribution to the disease.

Interestingly, serum 25-hydroxyvitamin D is a liposoluble vitamin whose concentration is decreased in humans and dogs with IBD for several reasons, including impaired absorption, fat malabsorption, restricted dietary intake, or reduced sunlight exposure [65,71]. Vitamin D supplementation has shown the ability to regulate gut microbiome and to decrease the intensity of intestinal inflammatory lesions in rodent models of IBD [72]. The effects of vitamin D on the gut microbiome appear to be mediated by the expression of the gene encoding cathelicidin antimicrobial peptide (CAMP) by epithelial cells and immune cells [73]. New research aiming at deciphering the effects of dietary vitamin D intake on the function of the microbiota and the possible beneficial effects on the evolution of IBD must be undertaken.

## 4. The Impact of Nutritional Interventions in Canine IBD

Approximately 50% of dogs with chronic inflammation are responsive to dietary changes and are considered to have Food Responsive Enteropathy (FRE) [74]. FRE is one of the most common forms of chronic inflammatory enteropathy in dogs and includes those with adverse food reactions (i.e., food allergy and food intolerance) and those with intestinal inflammation that benefits from properties of a different diet. Several nutritional interventions are being used to alleviate clinical signs. Highly digestible or hypoallergenic diets and industrial or home-prepared diets are promising candidates [75]. Prevailing large bowel diseases can also be managed with high-fiber diets [76]. Disorders comprising significant lymphangiectasia may be addressed with hyperdigestible low-fat diets [77].

### 4.1. Hypoallergenic Diets

Commercial hypoallergenic foods may have a role in managing canine IBD. Industrial hypoallergenic foods find their protein in plant sources (most often soybeans), insects, or bird feathers. Animal protein-free diets were shown to increase fecal bacterial richness and diversity in dogs with FRE compared to the control subjects. The fecal microbiota index, a PCR-based assay aiming at assessing the fecal microbiome by quantifying the abundance of predefined bacterial taxa, was significantly higher in dogs with FRE than in healthy controls. No significant differences in the composition of the gut microbiota were detected after the dietary trial [78]. Interestingly, a hydrolyzed protein diet with a probiotic strain *Enterococcus faecium* was associated with a significant increase in bacterial richness and improvement in clinical signs in dogs suffering from FRE [79]. Additionally, the intake of a novel protein diet with cod and rice was associated with significant changes in the abundance of several bacterial taxa, including higher abundances of the genera *Gemella* and *Peptococcus*, in dogs with FRE [80]. The evaluation of the effects of these diets seems to reveal a contribution to the modulation of the dysbiotic canine gut microbiota. Further studies are needed to evaluate and better understand the potential positive effects of such interventions in these dogs.

### 4.2. Fiber-Enriched Diets/Prebiotics

Prebiotics are defined as non-digestible food ingredients able to promote the growth of beneficial intestinal microorganisms. Fructooligosaccharides (FOS) were demonstrated to affect the gut microbiota in dogs with primary dysbiosis [81]. A diet supplemented with a prebiotic-rich fiber mixture composed of rice bran, banana flakes and deactivated yeasts was shown to be associated with lower abundances of sulfate-reducing bacteria from the order Desulfovibrionales and an increase in *Clostridium* clusters I and II in the fecal samples of dogs with IBD [82]. The addition of resistant starch, β-glucans, mannan oligosaccharides, and chondroitin sulfate to a hydrolyzed diet did not significantly improve the disease activity in dogs with IBD. However, the post-treatment histological score was significantly lower solely in dogs receiving fibers. From a functional perspective, dietary supplements were demonstrated to increase serum paraoxonase-1, total antioxidant capacity and cholesterol, despite the absence of significant differences between the fecal microbiota of dogs with and without fiber enrichment [83]. A metabolomic study underlined additional beneficial effects of prebiotics and glycosaminoglycans on lipid metabolism, and thereby on intestinal membrane integrity in dogs with IBD [84]. Alternatively, supplementation of a hydrolyzed diet with a brown seaweed (*Ascophyllum nodosum*) rich in fermentable fibers drove an increased abundance of the Ruminococcaceae and Rikenellaceae families and higher concentrations of acetate in the feces of dogs with IBD, without improving clinical signs [85]. Overall, these studies suggest that fiber-enriched diets/prebiotics may modulate the gut microbiota and ameliorate oxidative status in canine IBD (Figure 1). To date, no studies are available regarding the effects of highly digestible and low-fat industrial diets and homemade regimens on the gut microbiota of dogs with IBD.

### 4.3. Industrial or Home-Prepared Diets

Veterinarians and dog owners need to weigh up the available evidence when deciding whether to feed dogs with IBD a commercial or personalized home-prepared diet. According to equivalent macro-nutritional analysis, industrial kibble food differs from home-made diets by the incompressible starch content necessary for the extrusion process. While the authors agree that dog domestication was accompanied by a selection of genes encoding for proteins involved in starch digestion (pancreatic α-amylase 2B-AMY2B), there is still a debate on the individual variation of their expression and on the capabilities to digest starch [86,87]. Resistant starch escapes digestion and substantially impacts the composition of the gut microbiota, depending on the structure of the starches reaching the colon [88]. Many experimental rodent models of IBD document a reduction in inflammatory lesions during moderate starch supplementation compared to the animals fed a starch-free diet [89,90]. While the beneficial effects in humans are generally linked to enhanced butyrate production by bacterial fermentation, significant individual variations are observed. Therefore, it advocates for a personalized approach to starch intake origin, the host’s digestive abilities, and microbiome profile [91,92].

## 5. Conclusions

The pathogenesis of IBD in dogs is poorly characterized; however, recent evidence points to the interplay between diet and gut microbiota. Dietary interventions are likely to play a key role in the management of these diseases. Significant shifts in macronutrient composition, such as high-protein or high-fiber diets, have been associated with changes in the composition and function of the gut microbiota. Dietary fiber, starch, and protein content are known to contribute to such changes in microbiota and metabolome composition. A better understanding of the different dietary strategies available for dogs with IBD would help ensure the selection of the most appropriate diet. Therefore, considerable efforts should be applied to improving our knowledge of diet-microbiota–host molecular interactions. Similarly, further functional studies are required in order to gain mechanistic insights into this intricate loop in health and disease. This will help implement targeted and effective dietary interventions as a means to restoring health and mitigating microbiota-associated disorders, such as IBD. These evidence-based recommendations are increasingly imperative as the burden of inflammatory disorders increases in dogs. The prospect of designing such dietary interventions targeted specifically at increasing key bacterial metabolites to improve inflammatory status would be of interest, considering that formal dietary guidelines are lacking for subjects with IBD.
